# Tuning Beforehand: A Foresight on RNA Interference (RNAi) and In Vitro-Derived dsRNAs to Enhance Crop Resilience to Biotic and Abiotic Stresses

**DOI:** 10.3390/ijms22147687

**Published:** 2021-07-19

**Authors:** Eltayb Abdellatef, Nasrein Mohamed Kamal, Hisashi Tsujimoto

**Affiliations:** 1 Commission for Biotechnology and Genetic Engineering, National Center for Research, P.O. Box 2404, Khartoum 11111, Sudan; eltayb@ncr.gov.sd; 2 Arid Land Research Center, Tottori University, 1390 Hamasaka, Tottori 680-0001, Japan; renokamal@tottori-u.ac.jp; 3 Behavioural and Chemical Ecology Unit, International Centre of Insect Physiology and Ecology, P.O. Box 30772, Nairobi 00100, Kenya; 4 Agricultural Research Corporation, P.O. Box 30, Khartoum North 11111, Sudan

**Keywords:** RNAi, siRNA, dsRNA, miRNA, gene silencing, environmental risks, spray-induced gene silencing (SIGS), insects, risk, drought, heat, salinity

## Abstract

Crop yield is severely affected by biotic and abiotic stresses. Plants adapt to these stresses mainly through gene expression reprogramming at the transcriptional and post-transcriptional levels. Recently, the exogenous application of double-stranded RNAs (dsRNAs) and RNA interference (RNAi) technology has emerged as a sustainable and publicly acceptable alternative to genetic transformation, hence, small RNAs (micro-RNAs and small interfering RNAs) have an important role in combating biotic and abiotic stresses in plants. RNAi limits the transcript level by either suppressing transcription (transcriptional gene silencing) or activating sequence-specific RNA degradation (post-transcriptional gene silencing). Using RNAi tools and their respective targets in abiotic stress responses in many crops is well documented. Many miRNAs families are reported in plant tolerance response or adaptation to drought, salinity, and temperature stresses. In biotic stress, the spray-induced gene silencing (SIGS) provides an intelligent method of using dsRNA as a trigger to silence target genes in pests and pathogens without producing side effects such as those caused by chemical pesticides. In this review, we focus on the potential of SIGS as the most recent application of RNAi in agriculture and point out the trends, challenges, and risks of production technologies. Additionally, we provide insights into the potential applications of exogenous RNAi against biotic stresses. We also review the current status of RNAi/miRNA tools and their respective targets on abiotic stress and the most common responsive miRNA families triggered by stress conditions in different crop species.

## 1. Introduction

Various abiotic and biotic stresses decrease agricultural productivity. Biotic stresses caused by insects, nematodes, parasitic weeds; viral, bacterial, and fungal diseases constrain crop productivity and total agricultural production; in particular, viruses and pests cause a significant loss of plant productivity. The effective control of pests remains a challenge in various cropping systems. Plants are naturally immune to infections by most potential bacterial, viral, and fungal pathogens, but some of them can be extremely problematic to control. Conventional crop breeding methods have been used to develop cultivars resistant to various diseases, but they are time-consuming because of the limited availability of genetic resources of most crops. The emergence of new virulent strains of microorganisms that attack resistant cultivars illustrates the urgent need for the development of novel approaches to combat these highly variable crop pests.

**Figure 1:**
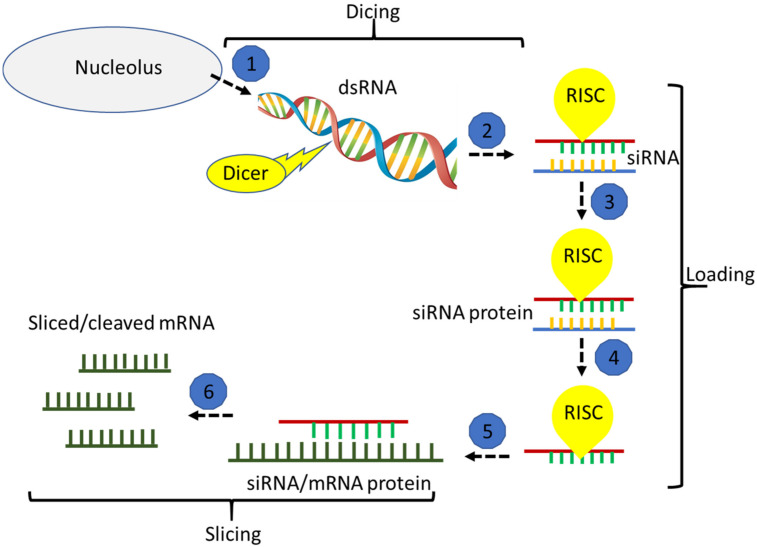
Illustration of the general RNAi pathway overview. Steps (1, 2) refer to dicing, (3, 4 and 5) refer to loading, (6) refer to slicing. (Adapted from [5,25]).

RNA interference (RNAi) is activated by double-stranded RNA (dsRNA); it is shared by most eukaryotes and plays a role in gene regulation and the defense against viral infections [1]. The applications of RNAi in agricultural fields largely depend on transformation approaches, where transgenic plants express dsRNAs that silence specific genes to control target traits [2]. Considering the potential effects of genetically modified (GM) organisms on ecological systems, the application of dsRNAs for gene silencing is a risk-free alternative [3,4]. RNAi is based on the expression of a dsRNA homologous to a target gene, which leads to the silencing of that gene. Figure 1 shows the three steps of breaking mRNA—the dicing step: dsRNA is sliced into siRNA duplexes by an RNase III Dicer; the loading step: siRNA is associated with an RNA-induced silencing complex (RISC); the slicing step: mRNA is degraded by RISC in a sequence-specific manner [5,6]. The technology has been used to study functional genomics of non-transformable species such as insects and nematodes [7]. The first step involves designing a dsRNA with a strand complementary to a fragment of the target gene. After checking to establish that the target gene mRNA level has been down-regulated, the study of the resultant phenotype illuminates the corresponding functions [8]. An example of such a study is the screening for receptor tyrosine kinase regulators in *Drosophila melanogaster* cells [9].

RNAi technology has been used against biotic stresses and abiotic stresses (drought, temperature, and salinity). Commercially, the technology has been used to engineer virus resistance in plants by expressing viral sequences as transgenes; for example, the coat protein from Papaya Ring Spot Virus was expressed in papaya, and the plants suppressed virus growth [10]. Silencing has been demonstrated for several insects and nematodes that were fed directly on diets containing the dsRNA of the target gene [11,12]. Host-induced gene silencing (HIGS), also described as host-induced RNAi by expressing dsRNA in plants, has shown promise in conferring resistance to insects, fungi, parasitic plants, and nematodes [13,14,15,16,17,18]. However, HIGS technology is limited because of the debates about transgenic plants in many countries and the lack of transformation protocols in many crop species [19]. Recent studies have shown that spray-induced gene silencing (SIGS) is very effective against insects, viruses, and fungi, and these studies provide solid evidence that fungi can devour external dsRNAs. Foliar application of dsRNA under greenhouse conditions in potato *Solanum tuberosum* L., against the Colorado potato beetle actin gene, provided increased resistance against this pest, and the resistance lasted for almost a month [20,21]. Wang et al. [19] showed that applying sRNAs or dsRNAs that target the necrotrophic fungal pathogen *Botrytis cinerea* Dicer-like1 and Dicer-like2 genes on the surface of fruits, vegetables, and flowers, significantly inhibited the growth of pathogens. This study confirmed the ability of *B. cinerea* to take up external sRNAs and long dsRNAs. In tobacco (*Nicotiana tabacum* L. cv. Xanthi), foliar spraying of dsRNA targeting the Tobacco Mosaic Virus (TMV) coat protein genes resulted in a 50–65% resistance to this virus [22]. 

Ref. [23] has demonstrated that foliar spraying of dsRNA (791 nt CYP3) on barley leaves, targeting the *Fusarium graminearum* ergosterol biosynthesis genes (CYP51A, CYP51B, CYP51C), inhibited *F. graminearum* growth locally (indirectly sprayed parts) and distally (in non-sprayed parts) in detached leaves. The efficient spray-induced control of fungal infections in the distal tissue involves the passage of CYP3-dsRNA via the plant vascular system and processing into small interfering RNAs (siRNAs) by fungal DICER-LIKE 1 (FgDCL-1) after uptake by the pathogen.

The phenomenon of RNAi has been studied in different abiotic environments, such as salinity, drought, extreme temperatures, heavy metals, nutrition deprivation, and radiation in various crops; temperature, drought, and salinity stresses cause larger crop losses than any other abiotic stress [24,25]. Numerous advancements in the analytical tools for biochemical, molecular, genomic, proteomic, and overall metabolic analysis are now allowing a better understanding of the complex regulatory network of stress-mediated responses.

Using such tools, the involvement of RNAi (small RNA species and their respective targets) in abiotic stress responses in many crops is well documented [26,27,28,29,30]. RNAi has been used effectively to incorporate abiotic stress tolerance traits in different crop species [31]. Despite the importance of RNAi technology and its implementations in agricultural systems through genetic transformation, there are safety concerns regarding long-term consequences for living organisms [32]. 

Current studies are mainly focused on expressing dsRNA and micro-RNAs (miRNAs) related to abiotic stress tolerance in crop plants. Spray-induced gene silencing (SIGS) is the most recent application of RNAi in agriculture. Spraying of dsRNA on plant leaves, stems, flowers, and fruits has the potential to serve as a smart solution to the public’s concerns about food safety issues, including genetically modified (GM) organisms and off-target risks associated with pesticide residues in crop plants. The targeted pathogens take up dsRNA and then use their RNAi machinery to amplify, process, and move on the silencing signal leading to silencing the targeted gene. Given the specific targeting and applicability to several plant’s biotic and abiotic stresses, the use of dsRNA and miRNAs offers a unique potential as a friendly agroecosystem protection strategy. In this review, we consider what is currently known and unknown about the roles of RNAi in plant responses to biotic and abiotic stresses and provide future prospects. We also discuss factors that affect the efficiency of SIGS applications in agriculture, the advantages and drawbacks of this technology, biosafety issues, and recent developments in producing insect-resistant crop plants. Finally, we highlight the importance and the roles of miRNAs families and their responses to different abiotic stresses and for different plant species. This information will later facilitate our investigation of the genetic basis of plant response and improve our understanding of enhancing plant performance under biotic and abiotic stress.

## 2. Vision One: Biotic Stress

### 2.1. Fate of DsRNAs in Soil and Soil-Living Organisms

**Figure 2:**
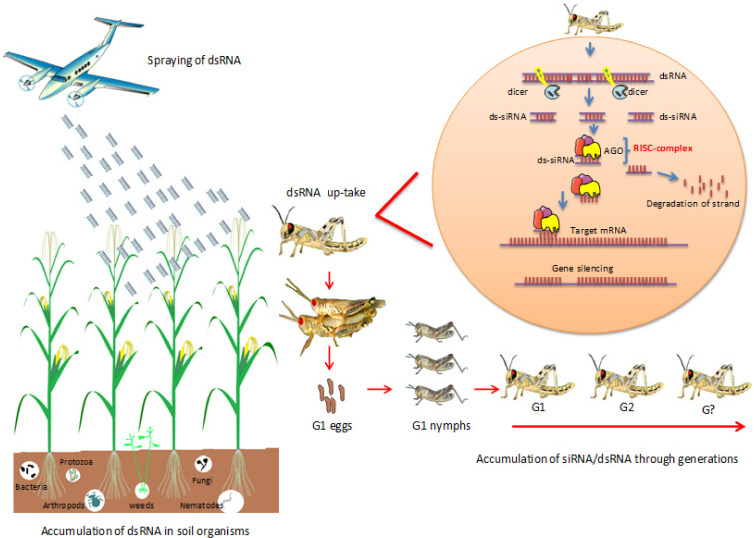
Predictable effect of dsRNA spraying on the long-term-insect transcriptome and soil living organisms’ behavior. The invasive insects take up the dsRNA directly or the plant-derived long dsRNA by sucking or chewing. In the insects’ cells, dsRNA process into 21 nt long siRNAs by the dicer (RNAse III enzyme). Produced siRNAs bind the RNAi-inducing silencing complex (RISC). The guide strand of siRNAs helps RISC to target the corresponding mRNA, leading to gene silencing and no protein expression. The siRNA silencing signals can remain for several generations.

In the field, sprayed dsRNA can accumulate in soil and be taken up by soil organisms such as nematodes, fungi, and bacteria (Figure 2). In a laboratory study, Dubelman et al. [33] analyzed the soil biodegradation of DvSnf7 dsRNA derived from a Monsanto GM maize, which confers resistance to corn rootworm (*Diabrotica* spp.). The authors used the QuantiGene assay to monitor DvSnf7 degradation and measured insect mortality to monitor its biological activity. DvSnf7 RNA (7.5 μg RNA/g soil) was degraded, and its biological activity was undetectable within approximately two days after application to three soil types, regardless of the pH or clay content. The authors reported that the DvSnf7 degradation rate did not depend on soil concentration within a range of 0.3–37.5 μg RNA/g soil. Accumulation of dsRNA in soil microorganisms has not been investigated, although fungi can take up external dsRNAs [19]. The nematode *Caenorhabditis elegans* can take up dsRNAs from the environment [34,35,36,37], and some herbivores can uptake dsRNAs with longer than 50–60 bp, but not sRNA [38,39]. Detailed studies are needed to follow the dsRNA uptake by soil organisms and to understand the genomic modifications that may occur (Figure 2). Future studies can be carried out using green fluorescent protein (GFP)-derived dsRNA (dsRNA-GFP) as a control to examine the persistence of dsRNA in soil-living organisms, and to investigate the long-term rotation of dsRNA between plants and pest or herbivore, corresponding with theories of the plant–pest interaction at the level of miRNAs and siRNAs, as well as the exposure to different dsRNA types in the same generation. No reports have indicated the accumulation of dsRNA/siRNA in fungal spores or parasitic plants (Figure 2). 

### 2.2. Long-Lasting Gene Silencing in Insects

The uptake of several dsRNAs targeting different genes with diverse sequences and the processing of these dsRNAs into 21-nt siRNAs by insect RNAi machinery, raise serious questions as to whether these thousands of siRNAs are capable of regulating gene expression in insects. Abdellatef et al. [18] has reported transgenerational silencing in the grain aphid *Sitobion avenae*; aphids feeding on transgenic barley expressing the salivary sheath protein (shp)-dsRNA showed morphological and physiological aberrations such as winged adults and delayed maturation over seven generations compared with those feeding on wild-type plants. Low expression of shp in aphids transferred from transgenic plants to wild-type plants for 1 or 2 weeks confirmed prolonged silencing [18]. Coleman et al. [40] reported the effect of RNAi on three generations of green peach aphids *Myzus persicae;* reproduction of aphids reared on dsMpC002 transgenic plants decreased by 60% compared with those reared on wild-type plants.

More studies are required to figure out the accumulation of dsRNA/siRNA through generations in different insect species and to expose these insects to different dsRNA sets during their life cycle. (Figure 2). Precautionary studies are needed to follow dsRNA/siRNA accumulation in predators such as ladybird beetles, spiders, and lacewings. 

### 2.3. Accumulation of SiRNA/DsRNA 

RNA uptake through diet and its possible impact on animals is an intriguing phenomenon and a highly discussed topic [41]. A pilot study demonstrated that plant small RNAs, acquired by animals through food intake, are delivered to specific organs and influence gene expression [42]. Several studies have elucidated the systemic spread of siRNA signals in plants [43,44]. Hunter et al. [45] exposed six-year-old citrus and grapevine trees to dsRNA by either root drenching, foliar spray, or trunk injections. The dsRNA could be detected for seven weeks after a single exposure to 2 g of dsRNA in 15 L.

The Asian citrus psyllid (*Diaphorina citri*), potato psyllid (*Bactericera cockerelli*), and the glassy-winged sharpshooter (*Homalodisca vitripennis*) took up the dsRNA after feeding on dsRNA-treated citrus trees. The dsRNA moved through the vascular system of the citrus trees, and the dsRNA was taken up, for instance, by psyllids that fed on the phloem. Moreover, the dsRNA was identified in psyllids and leafhoppers 5–8 days post-ingestion from plants, while in treated citrus tissues, dsRNA was found up to 57 days post-treatment [45]. 

**Figure 3:**
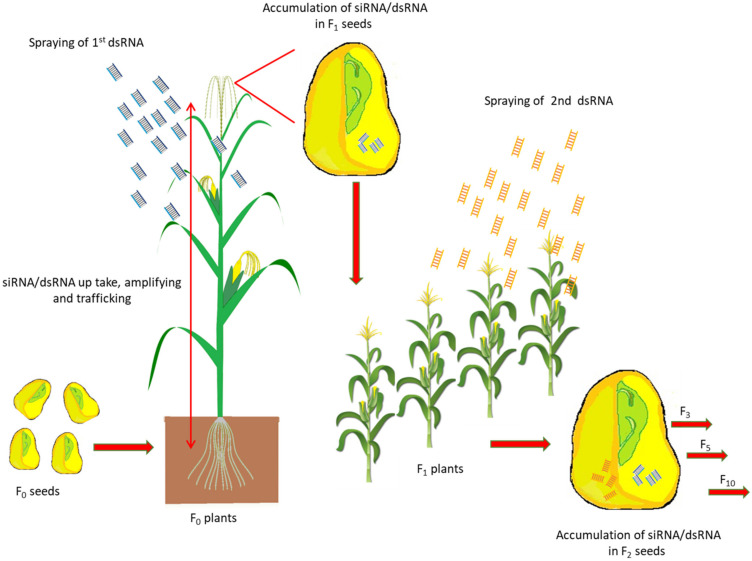
Predictable model illustrating effect of dsRNA spraying on seed genomic modifications. After foliar application of dsRNA, plants accumulate the Ex-dsRNA, and the dsRNA/siRNA signals in plants can spread systemically through the vascular tissue system. Accumulation of siRNA due to different applicable dsRNAs may lead to phenotypic variation in plants from generation to other generations. The expansion of siRNA could produce preferable mutations or modify the gene’s functions as the epigenetic modifications in plants can be directed and mediated by sRNAs.

A microinjection study in pumpkin, *Cucurbita maxima*, provided direct evidence that phloem small RNA binding protein 1 (CmPSRP1) mediates the trafficking among cells of 25-nt single-stranded RNA (siRNA), but not dsRNA [46]. The systemic spread of the silencing signal to the adjacent leaves within 1 h and the presence of dsRNAs up to 9 days post-application have been documented [22]. The efficient spray-induced control of *Fusarium graminearum* in the distal tissue involved the transport of CYP3-dsRNA via the plant vascular system and processing into siRNAs by FgDCL-1 after uptake by the fungi [23]. sRNAs are a core component of a signaling network that mediates epigenetic modifications in plants [47]. Epigenetic regulation can be mediated through a dynamic interplay between sRNAs, DNA methylation, and histone modifications, modulating transcriptional silencing of DNA. Regulatory sRNAs are short (20–24 nt), non-coding RNAs produced through the RNAi pathway that involves the plant-specific DNA-dependent RNA polymerases Pol IV and Pol V [48,49]. Overall, these examples suggest that siRNA or dsRNA could accumulate in seeds, and that exposure to different dsRNA doses may lead to genomic modifications in seed and plant; long-term experiments are needed to investigate the persistence of siRNA or dsRNA in seeds for several generations (Figure 2 and Figure 3).

## 3. Vision Two: Abiotic Stress

### 3.1. RNAi for Abiotic Stress Tolerance

**Figure 4:**
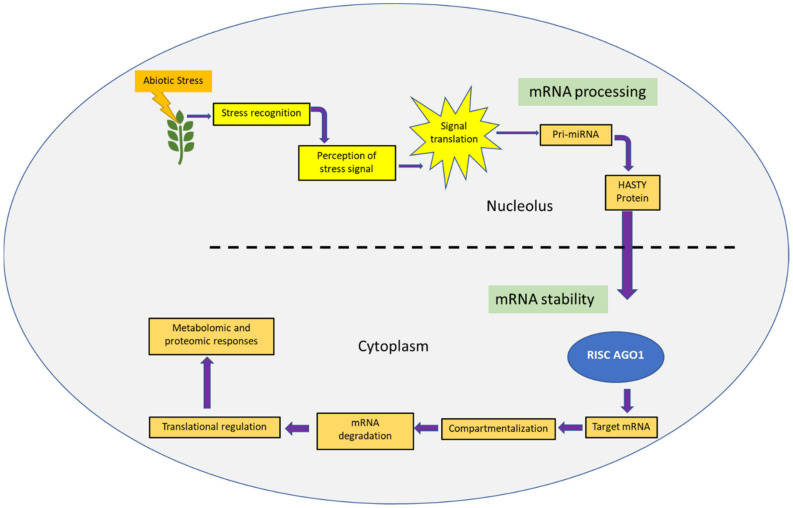
Pathway showing the steps involves in post transcriptional regulation mediated by abiotic stress-responsive miRNA genes in plants. Adapted from [4].

Abiotic stress tolerance in plants has developed as a responsive behavior to overcome sudden environmental changes and their negative effect on seed production. The RNAi strategy has been studied in different crops, and under various abiotic stresses, and it is proved to be a promising tool for crop breeding to abiotic stress using a post-transcriptional regulation [4,5,29,50,51]. RNAi has been efficaciously used to incorporate desired traits for abiotic stress tolerance in different crop species [31]. The rapid response and regulation of the gene expression allow plants to adapt their physiology to abiotic stresses. Basically, the gene expression is regulated at two main levels, the transcriptional and post-transcriptional level, where the regulations of gene expression occur at the levels of pre-messenger RNA (mRNA) processing (capping, splicing, and polyadenylation), mRNA stability, and mRNA translation [4] (Figure 4).

Most of the expression profiles of miRNAs that are involved in plant growth are significantly altered in response to abiotic stress environments. Here, we review the recent progress in RNAi and miRNA-mediated plant stress response/tolerance, and will discuss the main abiotic constraints for most crop production worldwide.

### 3.2. Drought Stress Tolerance

Drought affects plant growth and development, and many studies and examples have been reported. Wang et al. [52] found that the *AtHPR1* promoter driving an RNAi construct down-regulates farnesyltransferase in canola (*Brassica napus* L.) and protects yield under drought stress. Li et al. [53] created a transgenic rice line with the receptor for the activated C kinase 1 (RACK1) gene suppressed by RNAi; the drought tolerance of the GM rice is higher than that of a non-transgenic line. Jian et al. [54] reported that transgenic rice had a superior level of drought tolerance in contrast to non-transgenic rice plants. In relation to drought responses, the miR169g and miR393 genes were stimulated under drought conditions in rice [54,55] (Table 1). In peanuts (*Arachis hypogaea*), Zhao et al. [56] found that the GM peanut had a secure yield production and good quality under drought stress. It has also been reported that in drought-stressed rice, the miRNA expression profiling showed that many genes were drought-responsive genes that were down-regulated [51,57]. The expression patterns of miRNA for drought tolerance were explored in wild emmer wheat (*Triticum dicoccum*) and barley (*Hordeum vulgare*) by using the miRNA microarray platform [58]. In maize subjected to drought stress, up-regulation occurred in miR474, which interacts with proline dehydrogenase [59]. These authors concluded that drought stress up-regulated miR474 could down-regulate target gene PDH that negatively regulated proline accumulation to improve importation responses to drought stress [59].

MicroRNAs are important regulators of many development, environmental adaptation, and stress tolerance. miRNA-expression profiling under drought, salt, and cold stresses has now been performed in many plant species, including wheat, Arabidopsis, rice, barley, and maize (Table 1). Under drought, some of the miRNAs were shown to be responsive toward this stress in different plants. The miR393 function has contributed to antibacterial resistance through the inhibition of TIR1 expression to down-regulate auxin signaling and thereby the seedling growth under abiotic stress conditions [24]. The overexpression of miR393 led to less drought and salt tolerance in rice [60] (Table 1). Moreover, miR159 also responded to hormone signaling and dehydration responses in Arabidopsis [61,62]. In addition, several stress-related miRNAs have been identified in rice, and only two, miR393 and miR169 g (Table 1), were found to be related to abiotic stress; both were up-regulated by dehydration [56]. In transgenic rice, the expression of the receptor for the activated C kinase 1 (*RACK1*) gene was inhibited by RNAi, and this was used to elucidate the possible functions of *RACK1* in response to drought stress in rice [31,53]. The tolerance to drought stress of the transgenic rice plants was higher than non-transgenic rice plants. It was suggested that the *RACK1* decreases redox system-related tolerance to drought stress in rice plants [31]. In rice, a natural variation in the promoter of the transcription factor gene *OsLG3* is associated with and enhances drought-stress tolerance by inducing reactive oxygen species scavenging [63]. The functional analysis of *OsAHL1* revealed that the regulation of root development under drought conditions enhances drought avoidance. Furthermore, it participates in the oxidative stress response and regulates the chlorophyll content in rice. The authors of [64,65] found a decrease in the *BrDST71* gene expression in the drought-tolerant Chinese cabbage compared to the wild-type. They revealed that transgenic lines with the suppressed expression level of *BrDST71* had better drought tolerance than the wild-type and concluded that suppressing *BrDST71* gene expression was related to drought tolerance. Hu et al. [66] reported that RNAi-mediated suppression of *Oryza sativa GRXS17* (*OsGRXS17*) improved drought tolerance in rice.

Many other recent reports showed similar expression patterns of some miRNAs in different plants under stress condition [26] (Table 1). For example, miR1030 was involved in cold, salt in barley, and drought in rice (Table 1). It was reported that differential regulation of miRNAs differed in different plant tissues. In a study conducted on barley, four miRNAs displayed a tissue-specific regulation as a response to dehydration [67]. 

The expression profiles of miRNA in response to drought have been reported in many crop species; *Sorghum bicolor* [68]), *Gossypium hirsutum* [69]), *Oryza rufipogon* [70,71]), *Solanum tuberosum* [72]), *Triticum turgidum* [73]), *Hordeum vulgare* [74]), *Cucumis sativus* [75]), *Triticum aestivum* [76]), *Solanum lycopersicum* [77] and *Elettaria cardamomum* [29,78]).

Some miRNAs members, such as miR169, miR169g and miR169n, were up-regulated in rice [56]. Similarly, miR169 was also up-regulated in Arabidopsis under salt stress [56]. These reports demonstrate the variation in response patterns of the same miRNAs families across plants belonging to the same or different species under stress conditions (Table 1). Furthermore, it is also crucial for adaptation to stress to consider the tissue-specific regulation, which may be missed in the analyses of whole plants [79].

**Table 1:** Examples of RNAi and microRNAs applications in biotic and abiotic stress, their related references.

Target	Plant	Reference	
Tobamovirus, potyvirus, and alfamovirus	Tobacco	[80]	
Sugarcane mosaic virus SCMV	Maize	[81]	
Seed-borne mosaic virus (PSBMV)	Pea	[82]	
Cymbidium mosaic virus (CymMV)	Orchid	[83]	
Tobacco Mosaic Virus p126 replicase (TMV)	Tobacco	[22]	
Zucchini yellow mosaic virus (ZYMV)	Cucurbits	[84]	
Papaya ringspot virus CP	Papaya tree	[85]	
Fungi Fusarium graminearum	Barley	[26]	
Colorado potato beetle	Potato	[20]	
Plutella xylostella	Brassica	[86]	
Fungi Phakopsora pachyrhizi	Soybean	[87]	
Botrytis cinerea	Tomato, strawberry, iceberg lettuce, onion, and rose		[19]
**miRNA**	**Plant**	**Examples in Abiotic**	**Reference**
miR156	Arabidopsis	Heat	[88]
miR156, miR160, and miR164	Wheat	Drought	[89]
miR156, miR159, and miR160	Wheat	Heat	[89]
miR156, miR159 and miR319	Maize	Drought	[90]
miR157	Maize	Salinity	[91]
miR159	Wheat	Heat	[76]
	Rice	Heat/Cold	[92]
	Durum wheat	Drought	[93]
	Arabidopsis	Heat	[57]
	Rice	Cold	[57]
miR160	Rice	Heat	[94]
	Rice	Drought	[95]
	Rice	Salinity/Drought	[96]
	Wheat	Salinity	[97,98]
miR164, and miR1029	Wheat	Cold/Drought	[99]
miR166	Wheat	Drought	[93]
miR168, and miR474	Maize	Drought	[59,100]
miR169	Rice	Drought	[55]
	Arabidopsis	Drought	[101]
	Wheat	Heat/Cold	[102,103]
	Wheat	Heat	[104]
miR319	Rice	Cold	[105]
miR393, miR394, and miR164	Arabidopsis	Cold	[57]
miR393, miR166, and miR172	Wheat	Heat	[57,76]
miR396, and miR394	Rice	Drought/Heat	[92]
miR393	Arabidopsis	Salinity	[31]
	Rice	Salinity/Drought	[54,60]
miR394	Wheat	Drought	[106]
	Rice	Heat	[107]
miR408	Rice	Drought	[79]
	Wheat	Heat	[97]
miR529	Rice	Drought/Salinity	[92]
miR444	wheat	Salinity	[98]
	Barley	Salinity	[108,109]
miR827	Maize	Drought/Salinity	[100]
miR855	Wheat	Heat/Cold/Drought	[99]
miR5049	Durum wheat	Drought/Salinity	[93]
	Wheat	Salinity	[98]
miR5064	Barley	Drought	[110]
	Emmer wheat	Drought	[111]
miR1030	Barley	Drought	[112]
	Rice	Drought/Heat	[55,64,94]
	Barley	Cold/Salinity	[110]
	Wheat	Salinity	[112]

### 3.3. Salt Stress Tolerance

Salinity stress is a factor that affects crop production at both qualitative and quantitative levels [97,113,114]. The differential behavior of small RNAs has been observed in many plant species under salinity stress, on which the expression levels of miRNAs and other related genes are changed [27]. It was reported that the expression pattern of miRNAs under salt stress varies depending on the stress time course or duration. Several miRNA expression patterns were observed in the study of barley exposed to salt stress at different developmental growth stages. In this study, miR444 was up-regulated in the later part of stress duration but down-regulated after 8 and 27 h [108]. In rice, overexpression of miR444 induced the growth of the primary root and inhibited the lateral root growth [109]. In Arabidopsis exposed to various abiotic stress, the miR393 was up-regulated when exposed to higher salinity levels, dehydration, cold, and abscisic acid (ABA). Additionally, miR402, miR319c, miR397b, and miR389a were controlled by the level of abiotic stress in Arabidopsis [31]. A microarray study of maize was carried out to explain the miRNA profiles of a salt-tolerant and a salt-sensitive line. In maize roots under salt stress, the members of the miR396, miR156, miR167, and miR164 group were down-regulated, whereas the members of miR474, miR162, miR395, and miR168 groups were up-regulated [91].

In another study, [54] identified novel stress-related miRNAs from rice (*Oryza. sativa* L. ssp. *japonica* cv. 9522) seedlings subjected to cold, dehydration, salinity, and abscisic acid stresses as well as wild-type seedlings. Recent studies by Jagtap et al. [31] have shown that the C-kinase 1 activated receptor (*RACK1*) is a highly conserved scaffold protein with versatile functions and it plays important roles in the regulation of plant growth and development [31]. Transgenic rice plants, in which RNAi inhibited the expression of the *RACK1* gene, explained the possible functions of *RACK1* in response to drought stress in rice. The tolerance to drought stress of the transgenic rice plants was higher than non-transgenic rice plants. Li et al. [30] and Jagtap et al. [31] reported that OsRPK1 has an important role in the salt tolerance of rice; in the transgenic rice lines, the changes of proline content and the degree of cytoplasmic membrane damage were the main reasons for salt tolerance.

However, many researchers have found different results between plant species in response to abiotic stresses. The response of miR393, miR186, and miR156 has been up-regulated commonly in rice, wheat, and maize during drought and/or salt stress (Table 1). The miR169 was also reported to be induced by high salinity stress [115]. It was reported that the expression levels of miR156 family members (Table 1) were down-regulated considerably after salt shock in root tissue, as well as its involvement in drought response in wheat (Table 1) [91]. Based on these studies, it will be interesting to characterize miR444 and miR156 expression and function under salt stress conditions in other cereals. Despite these varying results, the up-regulation of miR393, miR160, miR169 and miR167 during drought and/or salt stress has been commonly observed in several plant species (Table 1). In contrast to the down-regulation of miR169 in Arabidopsis [101], drought stress up-regulated the expression of miR169 in rice (*Oryza sativa*) [56].

### 3.4. Tolerance to Stress Induced by Heat and Cold

Geographical conditions and seasonal deviations in temperature may decrease crop productivity. In response to inconsistent temperature variations, plants change their gene expression patterns at post-transcriptional levels [27]. Many miRNAs are induced in Arabidopsis under cold stress; some of them (miR398, miR394, and miR164) exhibit either transient or small responses under cold stress as reported by Liu et al. 2008 [57].

miRNAs in wheat varied in their expression responses to heat stress, using Solexa high-throughput sequencing. Thirty-two miRNA families were distinguished in wheat. Among them, nine miRNAs’ families were proposed as heat responsive: miR172 was distinctly decreased, whereas miRNAs such as miR166 and miR393 were up-regulated in response to heat stress [76].

Four wheat lines with gliadins down-regulated by RNAi have been characterized under heat stress and varying N availability, and silencing was found to be stable under heat stress [116]. Several temperature-responsive miRNA species have been identified in many plant species [117]. *Panicum virgatum* [118]), *Oryza sativa* [94,107,119], and *Triticum aestivum* [97] have explained the heat responsive changes in different miRNA species, whereas miRNAs, a chilling-responsive species, were characterized in *Glycine max* [120], *Zea mays* [94], and *Solanum habrochaites* [117].

As for the use of the application of dsRNA for enhancing crop physiology to combat abiotic stress, Kiselev et al. [121] analyzed the importance of some factors such as plant age, daytime, the causes of environmental stress, and the application of the dsRNA-induced suppression of the neomycin phosphotransferase II (*NPTII*) transgene. Their results showed that plant age, time of day, application means, and soil moisture were the factors affecting the exogenously induced transgene-silencing efficacy [122]. In Arabidopsis, [3] studied the possibility to influence the transgenes’ transcript levels as more prone sequences to silencing. These authors used a direct exogenous application of target long dsRNAs. Another study [121] revealed that synthesized dsRNAs designed to target the gene coding regions of enhanced green fluorescent protein (*EGFP*) or *NPTII* suppressed their transcript levels in Arabidopsis.

Several miRNAs belonging to different families were reported to be involved in cold-stress and heat stress responses. For example, miR156, miR159, miR166, and miR169, were up-regulated in Arabidopsis and wheat under cold and heat, while miR159 was down-regulated in rice under both conditions. The authors of [57,88,92,93] also reported that miR156 is responsible for heat stress memory in Arabidopsis [88]. Several miRNAs that were detected in response to heat and cold stress showed similar expression patterns. miR169 was characterized as a targeting nuclear transcription factor Y (NF-Y) in many plants, such as maize and Arabidopsis, which was up-regulated in wheat under both heat and cold stresses [102,103]. The processing of such miRNAs and their targets might improve the overall temperature stress tolerance of crop species. An investigation of post-transcriptional gene regulation by miRNAs under abiotic stress conditions is critical for a better understanding of stress tolerance in crop improvements. Although an extensive database on these studies exists, the quality of annotated miRNA entries is the most critical factor needed to refine its incompleteness and noise. These challenges also exist in other databases caused by composite factors, including the intrinsic defects of next-generation sequencing (NGS) methods, along with the limitation of miRNA annotation and noises and false positives resulting from different methods and standards Axtell [39]. Moreover, some conflicting results highlight the need for more in-depth and detailed characterizations of stress-responsive miRNAs in plants. Many methods have been described in RNAi applications; however, some limitations are related to their efficiencies and adoption. Both overexpression and artificial miRNA applications are effective techniques related to down-regulating or knock-out gene studies and easy methods to reveal miRNA function; however, a specific promoter is needed to improve their efficiency at the DNA level [123,124]. Overall, there is a lack of uniformity in the stress induction regimes applied by various research groups, making the comparisons of the responses among different reports difficult. Understanding the miRNA differential expression in other crop species will contribute to the direct application in the generation of drought, salinity, and heat-tolerant crops. Targeting a specific miRNA will provide evidence for the potential involvement of miRNAs in a broad range of stress response pathways.

## 4. Conclusions

RNAi-mediated epigenetic regulation is involved in most of the physiological and metabolic processes during plant growth and development. Recently, the application of SIGS has been presented as a powerful technique to replace GM crops, although the fate of sprayed dsRNA in the environment has been debated. Implementing this method will be accompanied by environmental risk assessments, which will consider the potential for harmful impacts on non-target organisms. Our current knowledge of the sensitivity of organisms to exposure to various doses of dsRNA is incomplete, and how they affect off-target genes (partially understood to some extent). Additional research addressing these areas is required to improve the certainty associated with risk assessments of RNAi applications in agriculture such as HIGS and SIGS. The possible incidence of dsRNA in the environment will certainly become an explosive field of investigation. This vision implies precise awareness from biotechnologists who intend to use dsRNA, especially in plant protection against pests and parasitic weeds. Furthermore, applications of RNAi technologies (HIGS and SIGS) would serve as an important approach for crop improvement, which will contribute significantly to crop productivity. There are numerous prospects for the use of RNAi in crop breeding for its enhancement, such as stress tolerance and enhanced nutritional levels. Thus, there is enormous potential in RNAi-based technologies for improving agricultural yield substantially. RNAi can be used against a variety of crucial gene targets in insects and pathogens. These studies collectively indicate the possibility of a positive effect of applying the RNAi technology to improve plant growth, development, nutritional value, and physiology. However, whether exogenous dsRNA-induced RNAi could efficiently mediate these roles is still largely unknown due to the lack of research in this area. The use of genomics approaches could help identify genes involved in dsRNA uptake. Extensive studies are needed to assure the RNAi-based plant protection strategies and related safety risks. The expression of several miRNA families in response to abiotic stresses has been detected. For example, miR156, miR159, 169, and miR393 families were reported as responsive to environmental conditions to regulate many stress-responsive mechanisms individually and/or together with their various miRNA partners. The availability of genome sequences will better facilitate manipulating these miRNAs, their targets, plant specificity, and plant stages will assess crop improvements under several abiotic stresses. Soon, RNAi technology will be one of the most promising solutions to enhance plant productivity as well as tolerance to stresses.

## Data Availability

Not applicable.
